# Mapping of Protein-Protein Interactions of *E. coli* RNA Polymerase with Microfluidic Mechanical Trapping

**DOI:** 10.1371/journal.pone.0091542

**Published:** 2014-03-18

**Authors:** Steven R. Bates, Stephen R. Quake

**Affiliations:** 1 Department of Applied Physics, Stanford University, Stanford, California, United States of America; 2 Department of Bioengineering and HHMI, Stanford University, Stanford, California, United States of America; Indian Institute of Science, India

## Abstract

The biophysical details of how transcription factors and other proteins interact with RNA polymerase are of great interest as they represent the nexus of how structure and function interact to regulate gene expression in the cell. We used an *in vitro* microfluidic approach to map interactions between a set of ninety proteins, over a third of which are transcription factors, and each of the four subunits of *E. coli* RNA polymerase, and we compared our results to those of previous large-scale studies. We detected interactions between RNA polymerase and transcription factors that earlier high-throughput screens missed; our results suggest that such interactions can occur without DNA mediation more commonly than previously appreciated.

## Introduction

High-throughput methods to map physical interactions among proteins and nucleic acids are of increasing prominence as tools in molecular biology [1], [2]. However, each method has its own limitations and biases, and different studies will each give their own maps of the interactome. Just as important as the mapping experiments themselves, therefore, is the emergence of comparative approaches that consider multiple distinct data sets covering the same interactors to build up a truer picture of the interactome than any single data set could provide [3], [4]. This comparative philosophy in turn implies that new methods of high-throughput mapping are of great value because of the independence of their biases from those of previously established methods.

**Figure 1:**
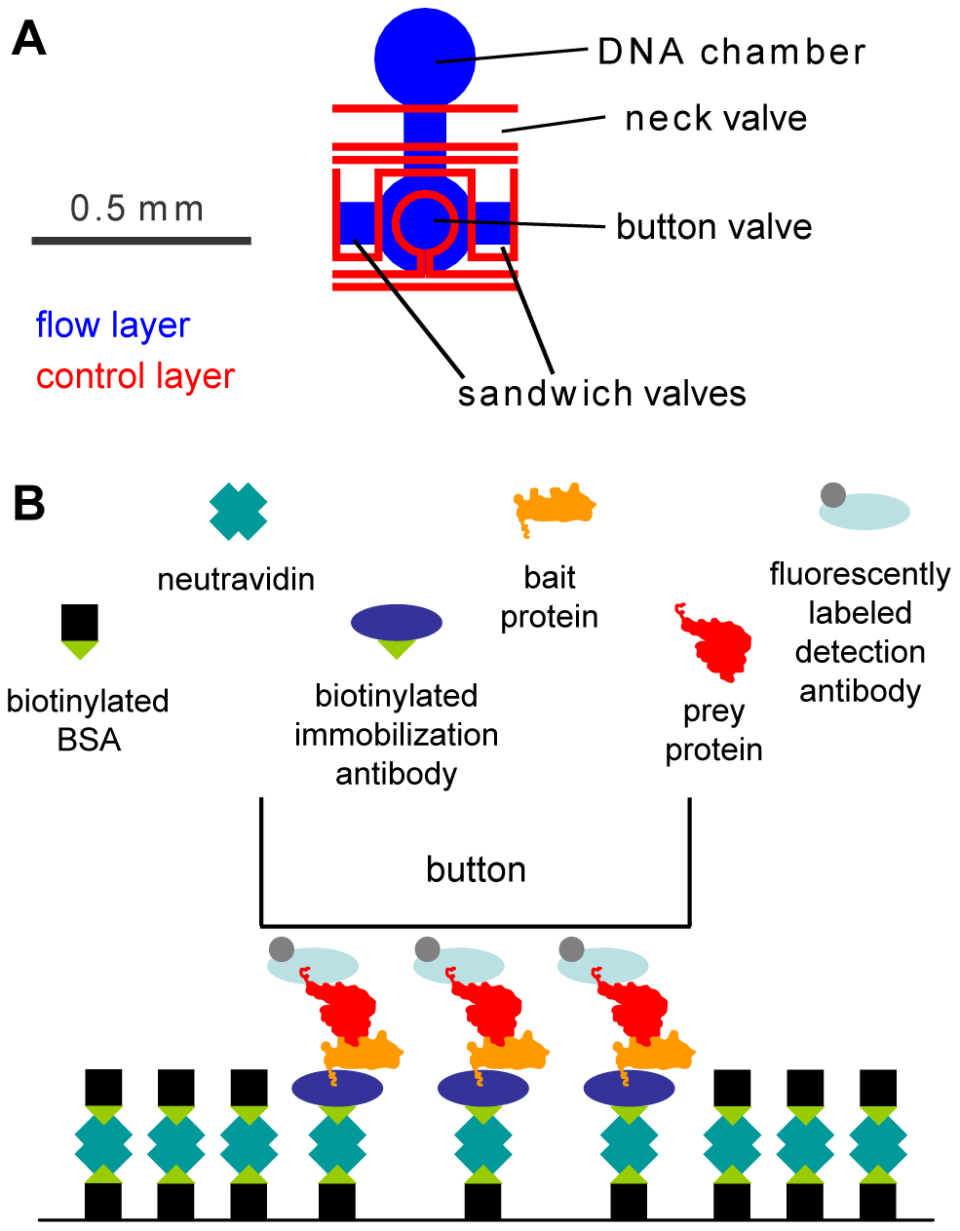
Principles of PING. (A) A single chamber of the PING chip. The sandwich valves are used to segregate adjacent chambers during ITT and equilibration after introduction of the labeling antibody. The DNA chamber contains the linear templates for the bait and prey to be expressed, and is closed off by the neck valve during all surface chemistry steps, prior to the ITT reaction. The button is used for mechanical trapping. (B) Cartoon of a mechanically-trapped interacting bait-prey pair after labeling, showing the layers of surface chemistry holding the bait in place.

One such recently developed method is a microfluidic technique called PING (protein-protein interaction network generation) [5], [6]. PING uses a polydimethylsiloxane (PDMS) microfluidic chip aligned on a glass slide over an array of DNA templates. Each pair of potentially interacting proteins is independently coexpressed in a segregated chamber of the chip by *in vitro* transcription and translation (ITT), with one of the potential interactors, the bait, being immobilized in a defined spot on the glass surface, and the other, the prey, being targeted by a labeled antibody that allows for fluorescent detection of interactions (Figure 1).

One of the central elements of a PING chip is the use of “buttons,” microfluidic valves that, by deflection of the PDMS membrane, cover part of the channel but do not block flow. Each chamber of the chip has a button, which is used for blocking during surface chemistry steps that define the analysis spots by depositing the bait immobilization antibody only under the button area, where it is held in place by the biotin-neutravidin interaction. Later, following the ITT reaction, the button holds the immobilized bait—along with any prey binding to it—in place during the subsequent wash steps and introduction of the labeling antibodies (Figure 1B).

We present a case study of protein-protein interactions involving the subunits of *E. coli* RNA polymerase. Our motivation was twofold: we compared the PING-generated network to previous work with the same potential interactors to explore how well PING did at capturing established interactions. At the same time, PING's unique strengths allowed for the detection of novel interactions not accessible by other high-throughput methods. In particular, yeast two-hybrid approaches are not well suited to transcription factors [7], [8], while affinity purification followed by mass spectrometry (AP-MS) requires one to perturb specific physiological states of the cell and has limited sensitivity. However, because of the nature of their function, mapping the network of transcription factor interactions is key to understanding the functional network of intracellular molecules. In particular, knowing the RNAP subunit contacted by a transcription factor is helpful for classifying the latter in terms of mode of action [9].

*E. coli* RNA polymerase is a multimeric protein complex consisting of two α subunits, one each of β, β′ and ω subunits, and a σ factor [10]. The σ factor is necessary for promoter recognition and transcription initiation, but during the transition from an initiation complex to a transcription elongation complex, it dissociates from the other subunits, which together constitute the core enzyme and transcribe all but the first ∼10 nucleotides on their own. *E. coli* has multiple σ factors, each specific to different sets of promoters and environmental conditions, and each capable of combining with the core enzyme to form RNA polymerase holoenzyme. The housekeeping σ factor used in expressing the majority of genes is the 70 kDa major σ factor, σ^70^. The role of the ω subunit is less clear. Though it has been shown to play roles in both complex assembly and transcription regulation, it is not strictly necessary for transcription [11], [12]. In this study we focus on interactions involving the three essential core enzyme subunits and the major σ factor, which will be referred to by their gene product names: rpoA (α), rpoB (β), rpoC (β′), and rpoD (σ^70^).

## Results

### Interaction screening experiments

We screened a total of 360 potential interactions: each of four RNAP subunit preys against 90 baits (including bait versions of each of the preys). We performed three trials, each trial interrogating the entire potential interaction set. Each trial was done in a PING chip with 2400 unit cells, which included six replicates expressing each bait-prey combination, 36 replicates expressing each prey without bait, one chamber expressing each bait without prey, and six chambers expressing neither bait nor prey (6×4×90+36×4+90+6 = 2400).

Baits were encoded with a C-terminal T7 epitope tag, and an N-terminal c-Myc epitope tag, while preys were encoded with C-terminal 6-His epitope tag. Baits were immobilized in the detection spots by a T7 epitope antibody, with bait expression levels measured by a Cy3-conjugated c-Myc epitope antibody, and interactions detected by an Alexa-Fluor-647-conjugated penta-His epitope antibody.

**Figure 2:**
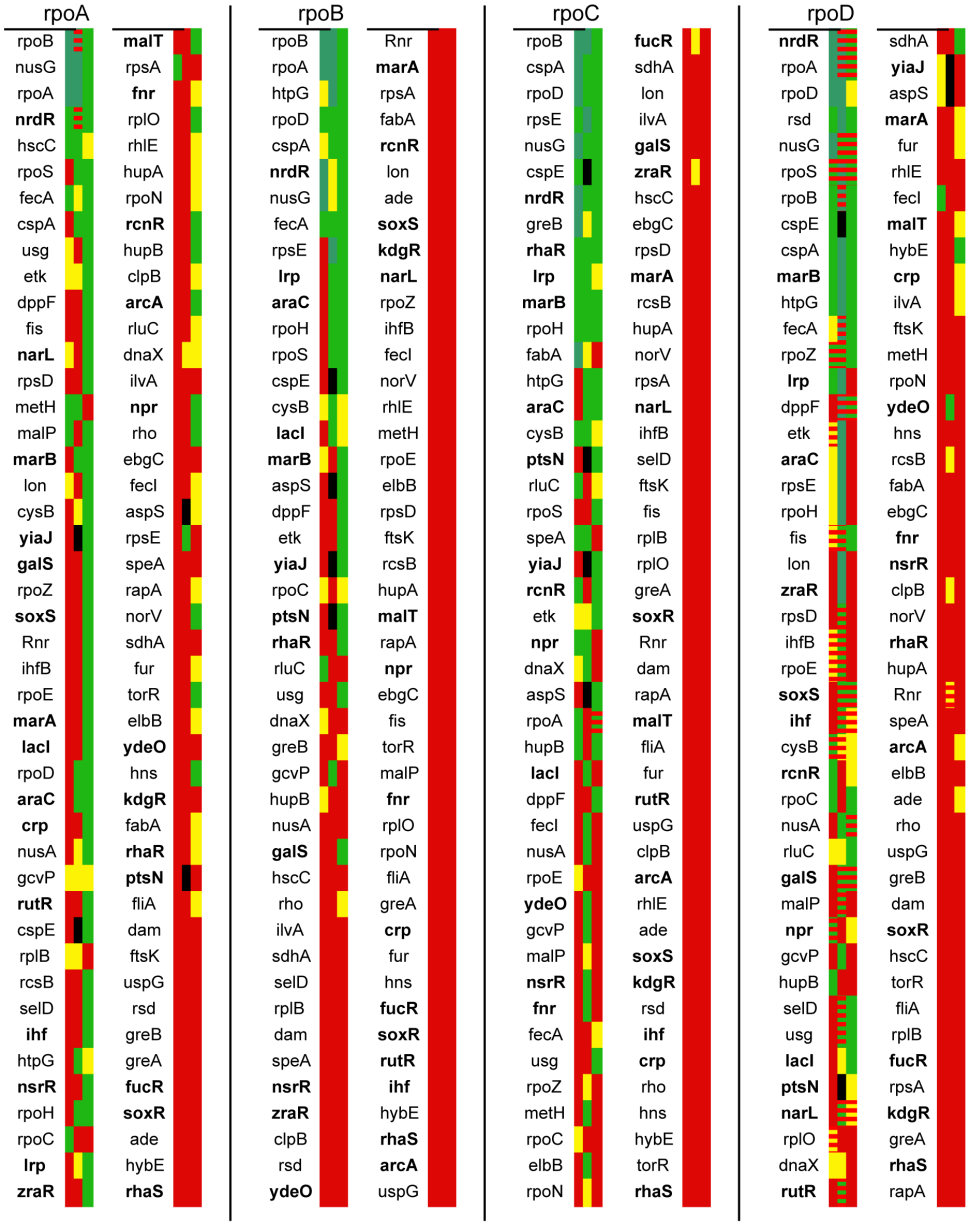
PING results across three screening experiments. For each prey, baits are ranked in ascending order of the average logarithm of p values, as an approximate metric of confidence in the interaction. (For each prey, the second column of baits continues from the first column, in increasing order of average log p.) Interactions with baits nearest the beginning of each list can be considered the best-established. The heat map represents the individual p values for each bait-prey combination across three experimental trials. Green indicates p≤0.01, with dark green indicating p<2×10^−5^, the limit of the computational hypothesis test (these were assigned an ad hoc p = 10^−6^ for ranking purposes). Yellow indicates 0.01<p≤0.05, and red background, hypothesis test failure. Red striping on a yellow or green background indicates that, although the interaction passed the hypothesis test comparing to baitless control spots, it was rejected due to high nonspecific background signal in the corresponding preyless spot. For the specific p values, see Table S2. Baits listed in bold are the set of twenty-two with no previously reported RNAP interactions, plus the five—crp, fnr, rhaR, rhaS, and soxS—which had previously reported interactions, but none identified in either of the two high-throughput AP-MS screens.

To establish the presence of an interaction, a computational hypothesis test was done to determine whether the median interaction signal from the set of replicates of each bait-prey combination was greater than the median interaction signal from the set of baitless chambers containing the appropriate prey. The resulting p values provided a metric of confidence of interaction and allowed for a better comparison across trials than would raw fluorescence data that would be susceptible to experiment-to-experiment variation (Figure 2).

Using the interaction signal from baitless spots as a baseline controlled for a major potential source of error: nonspecific binding, for instance between the interaction labeling antibody and the immobilization antibody, or between the preys and the immobilization antibody. The latter was most likely operative in the cases of the rpoB and rpoC preys, the baitless spots of which consistently gave a higher baseline interaction signal than those of rpoA and rpoD.

As a secondary nonspecific binding control to address the problem of “sticky” bait proteins as opposed to “sticky” prey proteins, for each bait-prey combination we calculated a bootstrap estimate of the 95% confidence interval for median interaction signal. If the lower bound of the interval was less than the interaction signal from the single preyless control spot with the appropriate bait, the interaction was rejected regardless of the p value from comparing to the baitless control set.

Carefully controlling for nonspecific binding was crucial to minimize false positives in this assay, but potential false negatives are a concern as well. In particular, some baits may simply fail to express, so in each experiment we measured expression levels of each bait with Cy3 fluorescence, applying the same hypothesis tests used to establish interactions, with the entire set of 150 baitless spots as a comparison baseline. Nine baits—cspA, cspE, ftsK, fur, greA, hupB, ihf, nrdR, and rcnR—failed to pass the expression test at the p≤0.05 level in any of the three experiments. However, several of these baits consistently passed the hypothesis test for interaction with one or more of the preys. This suggests that the expression labeling has insufficient sensitivity in these cases, and it is easy to imagine the reverse situation, an interaction where the expression labeling gives a true positive result but the interaction labeling fails to detect the binding.

In particular, interactions between rpoA and rpoC were not consistently detected nor were interactions between rpoC and rpoZ (the ω subunit), despite both interactions essentially being positive controls. It was because of this inconsistency that we did repeated trials, and considered an interaction to be detected if it passed the hypothesis test in at least one of the trials. However, the most appropriate way to consider the data is as a continuum of higher confidence to lower confidence interactions, which is roughly captured by ranking average log p.

There is one additional possible source of false positives specific to this study, but not inherent to PING. We considered binary interactions between individual RNAP subunits, which, in the case of the core subunits at least, would not exist as monomers physiologically. So there may be some interactions that represent true physical binding, but are biologically irrelevant because they involve surfaces of the subunits that are inaccessible in the enzyme complex. This hypothesis was ruled out for several interactions by independently validating against the full holoenzyme.

### Independent validation of selected PING-detected interactions

To confirm some of the interactions we discovered, we used intact, commercially-derived holoenzyme. We expressed *in vivo* and purified seven of the baits—npr, rcnR, nrdR, lrp, narL, rhaR, and zraR. We mixed each bait with holoenzyme at 6 μM bait and 1.11 μM RNAP, separated the enzyme from unbound bait by FPLC, and used mass spectrometry to analyze the fractions containing the holoenzyme for presence of the bait.

Four of the baits—lrp, narL, rhaR, and zraR—showed evidence of binding to the holoenzyme. Though npr, rcnR, and nrdR failed to show such evidence, this experiment does not absolutely rule out these interactions, as those baits may have dissociated too rapidly from the complex to be present in the collected RNAP fraction.

## Discussion

**Figure 3:**
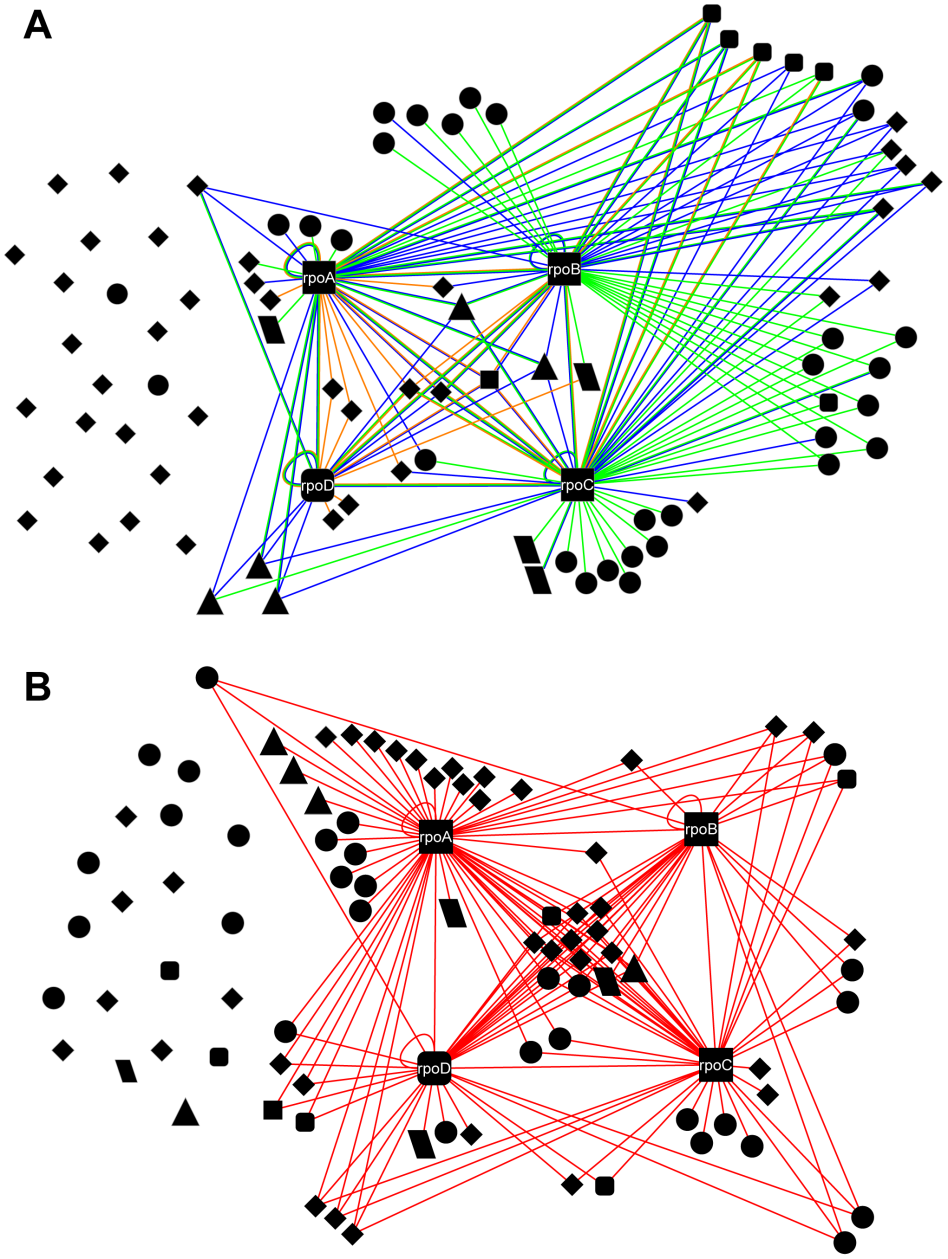
Two versions of the RNAP interaction network. (A) Previously-reported interactions. Green edges indicate interactions reported in the high-throughput study by Arifuzzaman et al., blue edges indicate interactions reported by Butland et al., and orange edges indicate interactions deposited in DIP or SwissProt with another reference [18], [20], [28]–[37]. Node shapes indicate the functional annotation for each protein. Squares correspond to RNAP subunits, with round-cornered squares being σ factors. Triangles correspond to ribosomal proteins, parallelograms to chaperones, and diamonds to transcription factors. All other proteins are represented by circles. (B) PING-generated map including interactions established at the p≤0.01 level in at least one trial. Though this map has approximately the same number of orphans (19) as the previously-reported map (22), they are not biased to transcription factors.

The pool of baits we chose to screen for interactions against the RNAP subunits fell into two categories. Sixty-eight were selected as previously discovered interactions by examining the protein interaction databases DIP and SwissProt for binding partners of the four preys. The largest sources of interactions deposited in these databases were two high-throughput studies, by Arifuzzaman *et al.* and Butland *et al.*, using AP-MS [13], [14]. However, Arifuzzaman *et al.* explicitly noted a failure to discover many expected RNA polymerase interactions with transcription factors, speculating that this was due either to low abundance of free (i.e. non-DNA bound) transcription factors, or to the transience of the interactions; the method is known to suffer from the aforementioned problem of maintaining a stable complex during purification [15]. Our pool includes several transcription factors that Arifuzzaman *et al.* reported as RNAP interactors, and several more that were identified as interactors by Butland *et al.* Nevertheless, the previously reported RNAP interaction network is notably deficient in transcription factors (Figure 3 A; Table S1). Accordingly, all but two of the twenty-two baits in the second category—proteins that had not been previously shown in a high-throughput screen to interact with RNAP, but would be expected to biologically—were transcription factors.

The buttons' mechanical trapping allows PING to capture interactions with fast dissociation rates that might be missed by other methods, and the ITT avoids the problem of failure to detect an interaction due to low cellular abundance of one of the partners. Previously published calibration experiments demonstrate that the method is capable of capturing interactions with affinities as weak as ∼1 μM [5]. PING should, therefore, be particularly well-suited to detecting interactions with transcription factors. Of particular interest are araC, fucR, galS, malT, and rhaS, which are major transcriptional regulators of sugar metabolism [16], as well as arcA, ihf, lrp, and narL, which are among the seven major transcription factors that together control transcription of the majority of *E. coli* genes [17]—the other three, crp, fnr, and fis, were known RNAP interactors from the databases, with fis being identified by Butland *et al.* as interacting with rpoC.

For the purposes of comparison to the previous high-throughput studies, we had to make a binary choice about whether each potential interaction was detected by our experiments. We chose to define an interaction as being detected by PING if it passed the interaction hypothesis test at the p≤0.01 level in at least one of the trials. However, there are other choices that could have been made, such as using a p≤0.05 cutoff instead of p≤0.01, requiring an interaction to pass the hypothesis test in at least two out of the three trials, or simply choosing a cutoff value for average log p. We stress, though, that it is most appropriate to consider the detection of interactions to fall on a continuum of confidence rather than being a simple yes-no question.

In addition to this type of quantitative analysis, the level of confidence of an interaction can also be evaluated by considering prior biological knowledge. For instance, previous work has shown that soxS, crp and ihf interact with RNAP specifically through the α subunit [18], [19], which our data confirms. Similarly rsd's function is to bind to the major sigma factor [20], and our results correctly show it to bind to rpoD but not to the other subunits. On the other hand, rhaS and crp have also been established as interacting with rpoD [19], [21], so our failure to detect this interaction is a false negative.

**Figure 4:**
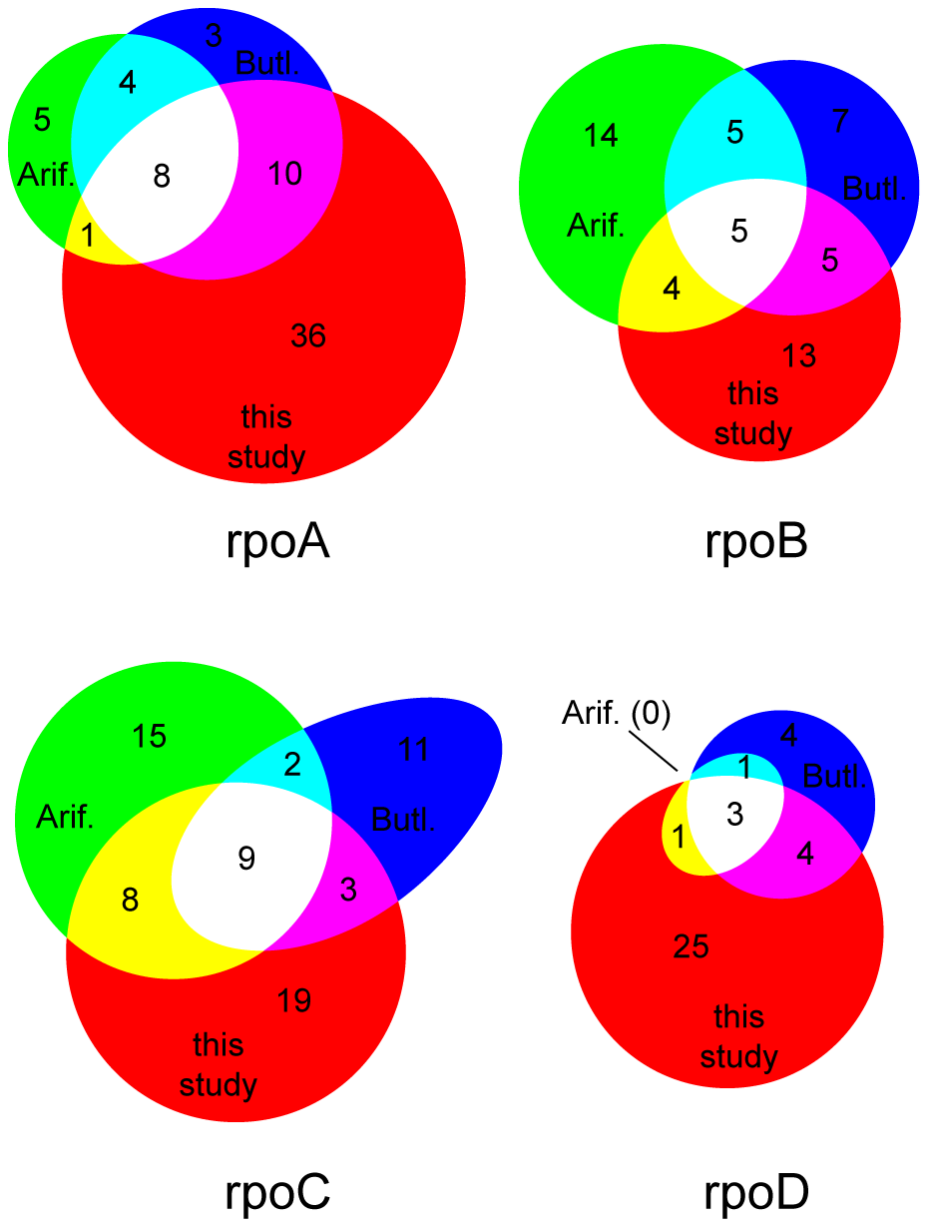
Comparison of PING results to previous high-throughput studies. For each prey, the corresponding Venn diagram gives the number of baits in our pool of 90 detected as interacting by Arifuzzaman *et al.*, Butland *et al.*, and by this study (p≤0.01 level in at least one trial).

Despite using the same method, the Arifuzzaman et al. and Butland et al. studies do not agree particularly well with one another, with ∼50% of the interactions reported by one study undetected by the other; the overlap between PING and either of the previous studies is nearly as good (Figure 4). This indicates that these previous studies suffer from a high false-positive or false-negative rate, or some combination of both. Though PING has its own sources of false-positive and false-negatives, the independence of the method strengthens the case for the previously-reported AP-MS interactions that we also captured, while the success rate of the FPLC-MS validation experiments indicates that the PING-only results include a large fraction of true positives. Therefore, we are able to report that we have validated 36 interactions that appeared in one but not the other AP-MS study, and have discovered 93 new potential interactions.

Our results for the seven major E. coli transcription factors—arcA, crp, fis, fnr, ihf, lrp, and narL—are striking: all bind to RNAP, and all except fnr to the α subunit (rpoA). Fnr binds to the β′ subunit (rpoC) instead, while fis binds additionally to the major σ factor (rpoD), and lrp to all subunits. Binding to only the α subunit is characteristic of Class I promoter activation, in which the transcription factor recognizes DNA sites upstream of the −35 element, and the only sterically feasible RNAP contact is the flexible C-terminal domain of the α subunit [17]. In contrast, Class II activation has the transcription factor binding near the −35 element, where it is accessible to the entire complex, although binding to the σ factor in addition to α is most typical for this category[19].

Our results allow us to determine whether the promoter sequence is required to mediate TF-RNAP binding. The accepted general model of transcription factor function is that the transcription factor binds first to the promoter sequence and then recruits RNA polymerase in the case of activation, or sterically blocks RNAP in the case of repression [17]. However, an alternative mechanism has been proposed [18], [22], [23] for some transcription factors— including marA and soxS—in which the TF binds to free RNAP first, then the TF-RNAP complex binds to DNA. One specific context for this mechanism is when the number of potential TF binding sites in the genome exceeds the expression level of the transcription factor; it is thought that in this case the transcription factor can more efficiently search the genome by first forming a complex with RNAP. In this manner, the complex binds only to sites in the genome that contain both the transcription factor binding sequence and sigma factor promoter recognition sequences.

The only DNA present in PING experiments is the expression templates, which are designed for T7 RNA polymerase and do not contain *E. coli* promoter sequences, while the FPLC-MS experiments do not include DNA at all. Under these promoter-free conditions, PING confirms both marA and soxS to be direct RNAP interactors through the α subunit.

However, the abundance of transcription factors in the PING-generated network (Figure 3B) suggests that many other TFs also interact directly with RNAP without the need for DNA mediation. This conclusion is further supported by the four interactors we validated under DNA-free conditions with FPLC-MS, despite the fact that lrp and narL have well-defined and highly specific consensus DNA-binding sequences [24], [25], and rhaR and zraR are also known to act by binding to promoter recognition sequences [26], [27]. We propose that binding to free RNAP without DNA may be a more general mode of action for *E. coli* transcription factors than previously appreciated. It is possible that TF-RNAP complexes frequently form before searching the DNA in order to raise the specificity of binding. Our results suggest a new direction of experimental inquiry exploring the functional implications of this possibility.

## Materials and Methods

### Expression template generation and PING device setup

Linear expression templates were generated by PCR from a library of Gateway clones. The PCR added coding sequences for N-terminal c-Myc epitope tag (EQKLISEEDL) and C-terminal T7 epitope tag (MASMTGGQQMG) to bait templates and for N-terminal His_6_ epitope tag to prey templates; start and stop codons and T7 promoter and terminator sequences were added to all clones. Templates were arrayed on Codelink epoxy slides (CEL Associates, Inc.), and the PDMS chip was aligned over the array and thermally bonded to the slide.

### Surface chemistry, ITT, and imaging

Surface chemistry reagents were introduced to the chip sequentially, with a phosphate-buffered saline wash following each step: biotinylated BSA (Pierce), neutravidin (Pierce), biotinylated BSA (with button valve closed), biotinylated T7-epitope antibody (EMD Biosciences; with button open). Then RTS 500 *E. coli* HY ITT reaction mix (Roche) with additional TNT T7 polymerase (Promega) added to boost transcription was introduced into the DNA chambers, and the chip was incubated for 2 hours at 30°C. Finally the labeling antibody mixture—Cy3-conjugated c-Myc epitope antibody (Sigma-Aldrich) and Alexa-Fluor-647-conjugated penta-His antibody (Qiagen)—was introduced, the chip was allowed to equilibrate 1 hour at room temperature, and the labeling mix was washed out with PBS. Imaging was done with either an Arrayworx (Applied Precision) or a TECAN LS Reloaded scanner, in both the Cy3 and Cy5 channels.

### Image and statistical analysis

The images were analyzed with GenePix Pro 6.0 (Molecular Devices). The background-subtracted signal for each spot in each channel was determined and used for all subsequent analysis. The computational hypothesis test for interactions was performed for each bait-prey pair on the Cy5 channel signal using MATLAB R2008a, by pooling the experimental data set (i.e. all replicates of the appropriate bait-prey combination, n = 6) and the control data set (i.e. the baitless spots expressing the appropriate prey, n = 36), randomly redistributing the pooled data points into a 6-member “simulated experimental” set and a 36-member “simulated control” set, finding the “simulated difference in medians” and repeating 50000 times to generate a probability distribution of difference in medians. Finally, a p value was determined by comparing the observed difference of experimental and control medians to the probability distribution. The hypothesis test for expression was performed analogously on the Cy3 channel signal, with n = 25 for the experimental data set, and n = 150 (all baitless spots, irrespective of prey) for the control data set.

### Interaction validation

Each of the seven baits selected for validation was cloned with a C-terminal His_6_ tag into a pET-28a(+) vector in One Shot BL21 (DE3) competent cells (Invitrogen), expressed, and purified with Ni-NTA resin (Invitrogen). Each bait was mixed with σ^70^ RNAP holoenzyme (Epicentre Biotechnologies) in 100 μl and run through a Superdex 200 10/300 GL column (GE Healthcare) with PBS as running buffer. The fractions containing the RNAP complex were submitted to the Stanford University Mass Spectrometry Facility for analysis. The fractions were trypsin digested and characterized by LC-MS/MS, and the peptide sequences were compared to the SwissProt database to identify the proteins in the fraction.

## Supporting Information

Table S1 Two versions of the RNAP interaction network. The pool of baits used in this study (each of the four RNAP subunit preys is present additionally as a bait) and their previously known interactions (compare to Figure 3; for the purposes of node shape codings, transcription factors include anti-σ factors and transcription elongation and termination factors). “Previously Reported Partners” indicates which of the four RNAP subunits α (A), β (B), β′ (C), or σ^70^ (D) each bait was previously known to bind to, and “Reference” indicates the source of the knowledge: Arifuzzaman *et al.*, Butland *et al.*, or another, low-throughput study cited by either the DIP or Swissprot database (db). “PING Detected Partners” indicates which subunits passed the interaction hypothesis test at the p≤0.01 level in at least one of the present study's trials.
(DOCX)

Table S2 PING results across three screening experiments. The p value of interaction for each combination of bait and prey, in each of the three experimental trials. Technical problems in the second experiment prevented the recovery of data for four baits—aspS, cspE, ptsN, and yiaJ—in the second experiment.
(DOCX)
