# Supplementary material for: Mapping of Protein-Protein Interactions of E. coli RNA Polymerase with Microfluidic Mechanical Trapping
Source: PLoS One. 2014 Mar 18;9(3):e91542. doi: 10.1371/journal.pone.0091542 (PMC3958368; doi:10.1371/journal.pone.0091542)
Supplement: Table S1 — Two versions of the RNAP interaction network. The pool of baits used in this study (each of the four RNAP subunit preys is present additionally as a bait) and their previously known interactions (compare to Figure 3; for the purposes of node shape codings, transcription factors include anti-σ factors and transcription elongation and termination factors). “Previously Reported Partners” indicates which of the four RNAP subunits α (A), β (B), β′ (C), or σ70 (D) each bait was previously known to bind to, and “Reference” indicates the source of the knowledge: Arifuzzaman et al., Butland et al., or another, low-throughput study cited by either the DIP or Swissprot database (db). “PING Detected Partners” indicates which subunits passed the interaction hypothesis test at the p≤0.01 level in at least one of the present study's trials. (DOCX) [file pone.0091542.s001.docx]

| Gene | Locus | PreviouslyReported Partners | Reference | PING Detected Partners | Function |
| --- | --- | --- | --- | --- | --- |
| rpoA | b3295 | ABCD | Arif(BCD),Butl(all),  db(all) | ABCD | RNAP |
| rpoB | b3987 | ABCD | Arif(ACD),Butl(all),  db(ACD) | ABCD | RNAP |
| rpoC | b3988 | ABCD | Arif(ABD),Butl(all),  db(ABD) | ABCD | RNAP |
| rpoD | b3067 | ABCD | Arif(ABC),Butl(all),  db(all) | ABCD | σ factor |
| ade | b3665 | B | Arif |  | nucleotide metabolism |
| araC | b0064 |  |  | ABCD | transcription factor |
| arcA | b4401 |  |  | A | transcription factor |
| aspS | b1866 | AC | Arif(C),Butl(A) | BC | tRNA synthesis |
| clpB | b2592 | C | Arif |  | chaperone |
| crp | b3357 | AD | db | A | transcription factor |
| cspA | b3556 | ABC | Arif(BC),Butl(A) | ABCD | transcription factor |
| cspE | b0623 | BC | Arif | ABCD | transcription factor |
| cysB | b1275 | AB | Butl(B),db(A) | ABC | transcription factor |
| dam | b3387 | BC | Arif |  | nucleotide metabolism |
| dnaX | b0470 | C | Arif | C | DNA polymerase |
| dppF | b3540 | B | Arif | ABC | membrane transport |
| ebgC | b3077 | A | Arif |  | carbohydrate metabolism |
| elbB | b3209 | B | Arif | C | lipid metabolism |
| etk | b0981 | A | Arif | ABCD | protein kinase |
| fabA | b0954 | BC | Arif | C | lipid metabolism |
| fecA | b4291 | C | Arif | ABD | membrane transport |
| fecI | b4293 | ABC | Arif(all),db(C) | CD | σ factor |
| fis | b3261 | AC | Butl(C),db(A) | AD | transcription factor |
| fliA | b1922 | ABC | Arif,db |  | σ factor |
| fnr | b1334 | AD | db | C | transcription factor |
| ftsK | b0890 | C | Arif |  | chromosome segregation |
| fucR | b2805 |  |  |  | transcription factor |
| fur | b0683 | A | Arif |  | transcription factor |
| galS | b2151 |  |  | AB | transcription factor |
| gcvP | b2903 | B | Arif | BCD | amino acid metabolism |
| greA | b3181 | ABC | Arif(A),Butl(all) |  | transcription elongation factor |
| greB | b3406 | ABC | Butl | C | transcription elongation factor |
| hns | b1237 | BC | Butl | A | transcription factor |
| hscC | b0650 | BD | Arif(B),db(D) | A | chaperone |
| htpG | b0473 | C | Arif,Butl | ABCD | chaperone |
| hupA | b4000 | ABC | Butl |  | transcription factor |
| hupB | b0440 | A | Butl | ACD | transcription factor |
| hybE | b2992 | A | Arif | D | chaperone |
| ihf | b1712 |  |  | A | transcription factor |
| ihfB | b0912 | C | Butl | A | transcription factor |
| ilvA | b3772 | C | Arif |  | amino acid metabolism |
| kdgR | b1827 |  |  |  | transcription factor |
| lacI | b0345 |  |  | ABCD | transcription factor |
| lon | b0439 | C | Arif | A | protease |
| lrp | b0889 |  |  | ABCD | transcription factor |
| malP | b3417 | BC | Arif(B),Butl(C) | A | carbohydrate metabolism |
| malT | b3418 |  |  | A | transcription factor |
| marA | b1531 |  |  | A | transcription factor |
| marB | b1532 |  |  | ABCD | transcription factor |
| metH | b4019 | C | Arif | AC | amino acid metabolism |
| narL | b1221 |  |  | A | transcription factor |
| norV | b2710 | B | Arif | A | riboflavin metabolism |
| npr | b3206 |  |  | AC | membrane transport |
| nrdR | b0413 |  |  | ABCD | transcription factor |
| nsrR | b4178 |  |  | AC | transcription factor |
| nusA | b3169 | ABCD | Arif(BC),Butl(all),  db(ABC) | ACD | transcription termination factor |
| nusG | b3982 | ABC | Arif,Butl | ABCD | transcription termination factor |
| ptsN | b3204 |  |  | BC | carbohydrate metabolism |
| rapA | b0059 | ABC | Arif(A),Butl(all) |  | helicase |
| rcsB | b2217 | A | Butl | A | signal transduction |
| rcnR | b2105 |  |  | ACD | transcription factor |
| rhaR | b3906 | D | db | BC | transcription factor |
| rhaS | b3905 | D | db |  | transcription factor |
| rhlE | b0797 | BC | Arif |  | RNA helicase |
| rho | b3783 | ABD | Arif(D),Butl(all) | A | transcription termination factor |
| rluC | b1086 | BC | Arif,But | BCD | rRNA synthesis |
| rnr | b4179 | BC | Arif | A | ribonuclease |
| rplB | b3317 | ABCD | Arif(ABC),Butl(all) |  | ribosomal |
| rplO | b3301 | ACD | Arif(A),Butl(all) | A | ribosomal |
| rpoE | b2573 | BC | Arif | AD | σ factor |
| rpoH | b3461 | ABC | Arif,Butl | ABCD | σ factor |
| rpoN | b3202 | ABC | Arif,Butl,db |  | σ factor |
| rpoS | b2741 | ABC | But | ABC | σ factor |
| rpoZ | b3649 | ABCD | Arif(B),Butl(all),  db(all) | AD | RNAP |
| rpsA | b0911 | ACD | Arif(C),Butl(AD) | A | ribosomal |
| rpsD | b3296 | ACD | Arif(A),Butl(all) | A | ribosomal |
| rpsE | b3303 | ABCD | Arif(A),Butl(all) | ABCD | ribosomal |
| rsd | b3995 | ABCD | Arif(CD),db(all) | D | anti-σ factor |
| rutR | b1013 |  |  | A | transcription factor |
| sdhA | b0723 | BC | Arif | D | carbohydrate metabolism |
| selD | b1764 | C | Arif | AD | amino acid metabolism |
| soxR | b4063 |  |  |  | transcription factor |
| soxS | b4062 | A | db | A | transcription factor |
| speA | b2938 | C | Arif | C | amino acid metabolism |
| torR | b0995 | BC | Butl | A | signal transduction |
| usg | b2319 | ABC | Arif(C),Butl(all) | ABCD | amino acid metabolism |
| uspG | b0607 | BC | Arif |  | universal stress protein |
| ydeO | b1499 |  |  | CD | transcription factor |
| yiaJ | b3574 |  |  | ABC | transcription factor |
| zraR | b4004 |  |  | AD | transcription factor |
