# Supplementary material for: Mapping of Protein-Protein Interactions of E. coli RNA Polymerase with Microfluidic Mechanical Trapping
Source: PLoS One. 2014 Mar 18;9(3):e91542. doi: 10.1371/journal.pone.0091542 (PMC3958368; doi:10.1371/journal.pone.0091542)
Supplement: Table S2 — PING results across three screening experiments. The p value of interaction for each combination of bait and prey, in each of the three experimental trials. Technical problems in the second experiment prevented the recovery of data for four baits—aspS, cspE, ptsN, and yiaJ—in the second experiment. (DOCX) [file pone.0091542.s002.docx]

| Bait | Prey | Trial 1 | Trial 2 | Trial 3 |
| --- | --- | --- | --- | --- |
| ade | rpoA: | 0.66 | 0.9999 | 0.63 |
|  | rpoB: | 0.74 | 1 | 0.37 |
|  | rpoC: | 0.96 | 1 | 0.45 |
|  | rpoD: | 0.99 | 0.9995 | 0.02 |
| araC | rpoA: | 0.4 | 0.005 | 0.01 |
|  | rpoB: | 0.11 | 0.00004 | 0.003 |
|  | rpoC: | 0.06 | 0.0001 | 0.003 |
|  | rpoD: | 0.03 | 0.000001 | 0.08 |
| arcA | rpoA: | 0.38 | 0.83 | 0.002 |
|  | rpoB: | 0.996 | 0.97 | 0.96 |
|  | rpoC: | 0.71 | 0.6 | 0.999 |
|  | rpoD: | 0.44 | 0.75 | 0.05 |
| aspS | rpoA: | 0.99 | N/A | 0.02 |
|  | rpoB: | 0.37 | N/A | 0.0006 |
|  | rpoC: | 0.38 | N/A | 0.002 |
|  | rpoD: | 0.049 | N/A | 0.11 |
| clpB | rpoA: | 0.19 | 0.11 | 0.03 |
|  | rpoB: | 0.96 | 0.16 | 0.83 |
|  | rpoC: | 0.99 | 0.44 | 0.94 |
|  | rpoD: | 0.55 | 0.049 | 0.27 |
| crp | rpoA: | 0.1 | 0.26 | 0.0009 |
|  | rpoB: | 0.96 | 0.88 | 0.92 |
|  | rpoC: | 0.91 | 0.64 | 0.95 |
|  | rpoD: | 0.72 | 0.14 | 0.02 |
| cspA | rpoA: | 0.4 | 0.006 | 0.00007 |
|  | rpoB: | 0.049 | 0.00004 | 0.00007 |
|  | rpoC: | 0.000001 | 0.00001 | 0.0003 |
|  | rpoD: | 0.005 | 0.000001 | 0.002 |
| cspE | rpoA: | 0.68 | N/A | 0.002 |
|  | rpoB: | 0.1 | N/A | 0.0002 |
|  | rpoC: | 0.0001 | N/A | 0.0002 |
|  | rpoD: | 0.002 | N/A | 0.00002 |
| cysB | rpoA: | 0.17 | 0.03 | 0.0007 |
|  | rpoB: | 0.03 | 0.0004 | 0.02 |
|  | rpoC: | 0.0007 | 0.002 | 0.03 |
|  | rpoD: | 0.007 | 0.03 | 0.02 |
| dam | rpoA: | 0.27 | 0.16 | 0.72 |
|  | rpoB: | 0.63 | 0.11 | 0.72 |
|  | rpoC: | 0.53 | 0.83 | 0.62 |
|  | rpoD: | 0.51 | 0.38 | 0.3 |
| dnaX | rpoA: | 0.46 | 0.047 | 0.03 |
|  | rpoB: | 0.046 | 0.14 | 0.26 |
|  | rpoC: | 0.03 | 0.006 | 0.09 |
|  | rpoD: | 0.02 | 0.02 | 0.49 |
| dppF | rpoA: | 0.054 | 0.13 | 0.00004 |
|  | rpoB: | 0.36 | 0.71 | 0.00004 |
|  | rpoC: | 0.42 | 0.27 | 0.0005 |
|  | rpoD: | 0.1 | 0.000001 | 0.01 |
| ebgC | rpoA: | 0.43 | 0.08 | 0.07 |
|  | rpoB: | 0.999 | 0.58 | 0.9996 |
|  | rpoC: | 0.55 | 0.08 | 0.84 |
|  | rpoD: | 0.27 | 0.08 | 0.29 |
| elbB | rpoA: | 0.4 | 0.6 | 0.03 |
|  | rpoB: | 0.9997 | 0.41 | 0.97 |
|  | rpoC: | 0.97 | 0.008 | 0.99 |
|  | rpoD: | 0.49 | 0.07 | 0.53 |
| etk | rpoA: | 0.02 | 0.04 | 0.0003 |
|  | rpoB: | 0.18 | 0.16 | 0.002 |
|  | rpoC: | 0.03 | 0.04 | 0.005 |
|  | rpoD: | 0.03 | 0.000001 | 0.07 |
| fabA | rpoA: | 0.65 | 0.77 | 0.02 |
|  | rpoB: | 0.67 | 0.7 | 0.45 |
|  | rpoC: | 0.000001 | 0.04 | 0.07 |
|  | rpoD: | 0.08 | 0.67 | 0.11 |
| fecA | rpoA: | 0.01 | 0.03 | 0.0001 |
|  | rpoB: | 0.01 | 0.00008 | 0.0006 |
|  | rpoC: | 0.2 | 0.26 | 0.046 |
|  | rpoD: | 0.03 | 0.000001 | 0.008 |
| fecI | rpoA: | 0.18 | 0.69 | 0.02 |
|  | rpoB: | 0.67 | 0.53 | 0.95 |
|  | rpoC: | 0.06 | 0.01 | 0.53 |
|  | rpoD: | 0.008 | 0.51 | 0.2 |
| fis | rpoA: | 0.18 | 0.08 | 0.00003 |
|  | rpoB: | 0.97 | 0.72 | 0.96 |
|  | rpoC: | 0.57 | 0.81 | 0.43 |
|  | rpoD: | 0.03 | 0.0002 | 0.007 |
| fliA | rpoA: | 0.994 | 0.99 | 0.03 |
|  | rpoB: | 0.995 | 0.76 | 0.9997 |
|  | rpoC: | 0.74 | 0.46 | 0.99993 |
|  | rpoD: | 0.43 | 0.52 | 0.81 |
| fnr | rpoA: | 0.13 | 0.12 | 0.02 |
|  | rpoB: | 0.98 | 0.76 | 0.96 |
|  | rpoC: | 0.37 | 0.003 | 0.83 |
|  | rpoD: | 0.11 | 0.33 | 0.18 |
| ftsK | rpoA: | 0.11 | 0.58 | 0.57 |
|  | rpoB: | 0.99 | 0.63 | 0.7 |
|  | rpoC: | 0.28 | 0.65 | 0.993 |
|  | rpoD: | 0.4 | 0.09 | 0.09 |
| fucR | rpoA: | 0.43 | 0.9 | 0.27 |
|  | rpoB: | 0.999 | 0.81 | 0.9998 |
|  | rpoC: | 0.36 | 0.04 | 0.999 |
|  | rpoD: | 0.61 | 0.45 | 0.86 |
| fur | rpoA: | 0.21 | 0.86 | 0.03 |
|  | rpoB: | 0.997 | 0.79 | 0.999 |
|  | rpoC: | 0.75 | 0.5 | 0.92 |
|  | rpoD: | 0.1 | 0.21 | 0.03 |
| galS | rpoA: | 0.093 | 0.11 | 0.0005 |
|  | rpoB: | 0.92 | 0.85 | 0.008 |
|  | rpoC: | 0.27 | 0.51 | 0.14 |
|  | rpoD: | 0.31 | 0.007 | 0.01 |
| gcvP | rpoA: | 0.035 | 0.02 | 0.04 |
|  | rpoB: | 0.94 | 0.005 | 0.45 |
|  | rpoC: | 0.98 | 0.0006 | 0.74 |
|  | rpoD: | 0.07 | 0.0004 | 0.93 |
| greA | rpoA: | 0.95 | 0.999 | 0.08 |
|  | rpoB: | 0.87 | 0.88 | 0.998 |
|  | rpoC: | 0.73 | 0.68 | 0.47 |
|  | rpoD: | 0.96 | 0.57 | 0.7 |
| greB | rpoA: | 0.63 | 0.99 | 0.1 |
|  | rpoB: | 0.16 | 0.57 | 0.02 |
|  | rpoC: | 0.000001 | 0.02 | 0.003 |
|  | rpoD: | 0.09 | 0.91 | 0.7 |
| hns | rpoA: | 0.97 | 0.95 | 0.009 |
|  | rpoB: | 0.998 | 0.93 | 0.86 |
|  | rpoC: | 0.96 | 0.73 | 0.92 |
|  | rpoD: | 0.09 | 0.4 | 0.14 |
| hscC | rpoA: | 0.002 | 0.00006 | 0.04 |
|  | rpoB: | 0.34 | 0.36 | 0.06 |
|  | rpoC: | 0.29 | 0.21 | 0.56 |
|  | rpoD: | 0.73 | 0.25 | 0.55 |
| htpG | rpoA: | 0.58 | 0.007 | 0.02 |
|  | rpoB: | 0.03 | 0.000001 | 0.0003 |
|  | rpoC: | 0.18 | 0.0001 | 0.0005 |
|  | rpoD: | 0.01 | 0.000001 | 0.01 |
| hupA | rpoA: | 0.19 | 0.12 | 0.02 |
|  | rpoB: | 0.97 | 0.49 | 0.9996 |
|  | rpoC: | 0.24 | 0.41 | 0.9991 |
|  | rpoD: | 0.08 | 0.33 | 0.52 |
| hupB | rpoA: | 0.97 | 0.9 | 0.0007 |
|  | rpoB: | 0.04 | 0.79 | 0.17 |
|  | rpoC: | 0.01 | 0.33 | 0.008 |
|  | rpoD: | 0.001 | 0.56 | 0.06 |
| hybE | rpoA: | 0.99 | 1 | 0.53 |
|  | rpoB: | 0.96 | 1 | 0.95 |
|  | rpoC: | 0.99 | 0.9999 | 0.66 |
|  | rpoD: | 0.999 | 0.9998 | 0.002 |
| ihf | rpoA: | 0.55 | 0.57 | 0.0002 |
|  | rpoB: | 0.99 | 0.93 | 0.97 |
|  | rpoC: | 0.74 | 0.83 | 0.88 |
|  | rpoD: | 0.13 | 0.0006 | 0.04 |
| ihfB | rpoA: | 0.59 | 0.13 | 0.00015 |
|  | rpoB: | 0.9 | 0.76 | 0.47 |
|  | rpoC: | 0.74 | 0.65 | 0.3 |
|  | rpoD: | 0.03 | 0.0003 | 0.12 |
| ilvA | rpoA: | 0.14 | 0.09 | 0.1 |
|  | rpoB: | 0.55 | 0.09 | 0.41 |
|  | rpoC: | 0.29 | 0.06 | 0.93 |
|  | rpoD: | 0.76 | 0.14 | 0.02 |
| kdgR | rpoA: | 0.57 | 0.06 | 0.29 |
|  | rpoB: | 0.42 | 0.78 | 0.92 |
|  | rpoC: | 0.78 | 0.69 | 0.92 |
|  | rpoD: | 0.98 | 0.28 | 0.98 |
| lacI | rpoA: | 0.7 | 0.12 | 0.0002 |
|  | rpoB: | 0.8 | 0.00002 | 0.03 |
|  | rpoC: | 0.89 | 0.001 | 0.06 |
|  | rpoD: | 0.11 | 0.045 | 0.01 |
| lon | rpoA: | 0.02 | 0.07 | 0.002 |
|  | rpoB: | 0.83 | 0.71 | 0.45 |
|  | rpoC: | 0.89 | 0.2 | 0.09 |
|  | rpoD: | 0.19 | 0.000001 | 0.25 |
| lrp | rpoA: | 0.65 | 0.02 | 0.01 |
|  | rpoB: | 0.06 | 0.00005 | 0.003 |
|  | rpoC: | 0.0001 | 0.00004 | 0.043 |
|  | rpoD: | 0.01 | 0.000001 | 0.07 |
| malP | rpoA: | 0.002 | 0.09 | 0.01 |
|  | rpoB: | 0.97 | 0.85 | 0.85 |
|  | rpoC: | 0.25 | 0.02 | 0.09 |
|  | rpoD: | 0.33 | 0.001 | 0.07 |
| malT | rpoA: | 0.45 | 0.17 | 0.002 |
|  | rpoB: | 0.98 | 0.67 | 0.73 |
|  | rpoC: | 0.99 | 0.34 | 0.9993 |
|  | rpoD: | 0.11 | 0.35 | 0.04 |
| marA | rpoA: | 0.52 | 0.16 | 0.0002 |
|  | rpoB: | 0.99 | 0.18 | 0.92 |
|  | rpoC: | 0.52 | 0.13 | 0.79 |
|  | rpoD: | 0.1 | 0.13 | 0.04 |
| marB | rpoA: | 0.87 | 0.01 | 0.0003 |
|  | rpoB: | 0.04 | 0.12 | 0.0002 |
|  | rpoC: | 0.0002 | 0.0002 | 0.01 |
|  | rpoD: | 0.01 | 0.000001 | 0.002 |
| metH | rpoA: | 0.002 | 0.007 | 0.06 |
|  | rpoB: | 0.91 | 0.41 | 0.999 |
|  | rpoC: | 0.53 | 0.009 | 0.9997 |
|  | rpoD: | 0.07 | 0.1 | 0.53 |
| narL | rpoA: | 0.023 | 0.22 | 0.0001 |
|  | rpoB: | 0.99 | 0.38 | 0.84 |
|  | rpoC: | 0.54 | 0.42 | 0.6 |
|  | rpoD: | 0.45 | 0.008 | 0.02 |
| norV | rpoA: | 0.68 | 0.83 | 0.007 |
|  | rpoB: | 0.999 | 0.85 | 0.4 |
|  | rpoC: | 0.37 | 0.39 | 0.75 |
|  | rpoD: | 0.29 | 0.43 | 0.08 |
| npr | rpoA: | 0.82 | 0.97 | 0.002 |
|  | rpoB: | 0.97 | 0.58 | 0.9993 |
|  | rpoC: | 0.004 | 0.01 | 0.4 |
|  | rpoD: | 0.001 | 0.6 | 0.04 |
| nrdR | rpoA: | 0.0001 | 0.007 | 0.00005 |
|  | rpoB: | 0.000001 | 0.03 | 0.009 |
|  | rpoC: | 0.000001 | 0.003 | 0.001 |
|  | rpoD: | 0.000001 | 0.000001 | 0.00004 |
| nsrR | rpoA: | 0.88 | 0.47 | 0.0002 |
|  | rpoB: | 0.99 | 0.06 | 0.98 |
|  | rpoC: | 0.68 | 0.001 | 0.997 |
|  | rpoD: | 0.27 | 0.07 | 0.37 |
| nusA | rpoA: | 0.1 | 0.03 | 0.008 |
|  | rpoB: | 0.28 | 0.08 | 0.26 |
|  | rpoC: | 0.16 | 0.004 | 0.51 |
|  | rpoD: | 0.08 | 0.009 | 0.01 |
| nusG | rpoA: | 0.000001 | 0.000001 | 0.00003 |
|  | rpoB: | 0.000001 | 0.03 | 0.01 |
|  | rpoC: | 0.000001 | 0.0001 | 0.002 |
|  | rpoD: | 0.000001 | 0.00006 | 0.00055 |
| ptsN | rpoA: | 0.98 | N/A | 0.07 |
|  | rpoB: | 0.61 | N/A | 0.005 |
|  | rpoC: | 0.08 | N/A | 0.0009 |
|  | rpoD: | 0.08 | N/A | 0.02 |
| rapA | rpoA: | 0.22 | 0.39 | 0.04 |
|  | rpoB: | 0.92 | 0.57 | 0.993 |
|  | rpoC: | 0.66 | 0.51 | 0.994 |
|  | rpoD: | 0.18 | 15 | 0.43 |
| rcnR | rpoA: | 0.59 | 0.89 | 0.001 |
|  | rpoB: | 0.39 | 0.7 | 0.9 |
|  | rpoC: | 0.002 | 0.5 | 0.004 |
|  | rpoD: | 0.0009 | 0.12 | 0.04 |
| rcsB | rpoA: | 0.82 | 0.36 | 0.0002 |
|  | rpoB: | 0.999 | 0.46 | 0.99 |
|  | rpoC: | 0.61 | 0.13 | 0.92 |
|  | rpoD: | 0.32 | 0.02 | 0.91 |
| rhaR | rpoA: | 0.95 | 0.93 | 0.02 |
|  | rpoB: | 0.07 | 0.47 | 0.006 |
|  | rpoC: | 0.0002 | 0.001 | 0.0005 |
|  | rpoD: | 0.18 | 0.67 | 0.09 |
| rhaS | rpoA: | 0.997 | 1 | 0.91 |
|  | rpoB: | 0.93 | 0.9996 | 0.99 |
|  | rpoC: | 0.997 | 0.999 | 1 |
|  | rpoD: | 0.999 | 0.9997 | 0.96 |
| rhlE | rpoA: | 0.2 | 0.1 | 0.02 |
|  | rpoB: | 0.74 | 0.51 | 0.93 |
|  | rpoC: | 0.83 | 0.52 | 0.999 |
|  | rpoD: | 0.15 | 0.07 | 0.07 |
| rho | rpoA: | 0.49 | 0.5 | 0.009 |
|  | rpoB: | 0.989 | 0.65 | 0.02 |
|  | rpoC: | 0.98 | 0.6 | 0.993 |
|  | rpoD: | 0.2 | 0.25 | 0.4 |
| rluC | rpoA: | 0.19 | 0.17 | 0.02 |
|  | rpoB: | 0.004 | 0.25 | 0.25 |
|  | rpoC: | 0.0001 | 0.35 | 0.02 |
|  | rpoD: | 0.03 | 0.046 | 0.009 |
| rnr | rpoA: | 0.053 | 0.08 | 0.002 |
|  | rpoB: | 0.91 | 0.2 | 0.88 |
|  | rpoC: | 0.69 | 0.38 | 0.97 |
|  | rpoD: | 0.74 | 0.03 | 0.67 |
| rplB | rpoA: | 0.02 | 0.047 | 0.06 |
|  | rpoB: | 0.13 | 0.71 | 0.45 |
|  | rpoC: | 0.22 | 0.96 | 0.97 |
|  | rpoD: | 0.43 | 0.59 | 0.88 |
| rplO | rpoA: | 0.17 | 0.24 | 0.009 |
|  | rpoB: | 0.97 | 0.74 | 0.9994 |
|  | rpoC: | 0.55 | 0.38 | 0.99 |
|  | rpoD: | 0.01 | 0.12 | 0.09 |
| rpoA | rpoA: | 0.000001 | 0.000001 | 0.00003 |
|  | rpoB: | 0.000001 | 0.000001 | 0.00004 |
|  | rpoC: | 0.01 | 0.55 | 0.004 |
|  | rpoD: | 0.000001 | 0.000001 | 0.00004 |
| rpoB | rpoA: | 0.000001 | 0.000001 | 0.00002 |
|  | rpoB: | 0.000001 | 0.000001 | 0.00003 |
|  | rpoC: | 0.000001 | 0.000001 | 0.0002 |
|  | rpoD: | 0.0003 | 0.000001 | 0.004 |
| rpoC | rpoA: | 0.006 | 0.08 | 0.21 |
|  | rpoB: | 0.03 | 0.16 | 0.02 |
|  | rpoC: | 0.05 | 0.7 | 0.2 |
|  | rpoD: | 0.001 | 0.63 | 0.01 |
| rpoD | rpoA: | 0.26 | 0.01 | 0.007 |
|  | rpoB: | 0.002 | 0.00004 | 0.0004 |
|  | rpoC: | 0.000001 | 0.0001 | 0.0002 |
|  | rpoD: | 0.000001 | 0.000001 | 0.02 |
| rpoE | rpoA: | 0.68 | 0.18 | 0.0001 |
|  | rpoB: | 0.98 | 0.4 | 0.96 |
|  | rpoC: | 0.02 | 0.08 | 0.22 |
|  | rpoD: | 0.04 | 0.0003 | 0.24 |
| rpoH | rpoA: | 0.89 | 0.01 | 0.01 |
|  | rpoB: | 0.11 | 0.00006 | 0.002 |
|  | rpoC: | 0.0005 | 0.0002 | 0.006 |
|  | rpoD: | 0.03 | 0.000001 | 0.73 |
| rpoN | rpoA: | 0.15 | 0.17 | 0.02 |
|  | rpoB: | 0.997 | 0.92 | 0.8 |
|  | rpoC: | 0.38 | 0.04 | 0.84 |
|  | rpoD: | 0.13 | 0.32 | 0.1 |
| rpoS | rpoA: | 0.06 | 0.006 | 0.00003 |
|  | rpoB: | 0.19 | 0.004 | 0.00003 |
|  | rpoC: | 0.19 | 0.06 | 0.0002 |
|  | rpoD: | 0.004 | 0.000001 | 0.00004 |
| rpoZ | rpoA: | 0.3 | 0.09 | 0.0002 |
|  | rpoB: | 0.92 | 0.51 | 0.68 |
|  | rpoC: | 0.65 | 0.04 | 0.15 |
|  | rpoD: | 0.0002 | 0.0005 | 0.004 |
| rpsA | rpoA: | 0.005 | 0.07 | 0.6 |
|  | rpoB: | 0.29 | 0.74 | 0.83 |
|  | rpoC: | 0.15 | 0.86 | 0.9 |
|  | rpoD: | 0.52 | 0.82 | 0.57 |
| rpsD | rpoA: | 0.053 | 0.11 | 0.0001 |
|  | rpoB: | 0.8 | 0.77 | 0.66 |
|  | rpoC: | 0.14 | 0.52 | 0.64 |
|  | rpoD: | 0.09 | 0.00004 | 0.2 |
| rpsE | rpoA: | 0.6 | 0.008 | 0.61 |
|  | rpoB: | 0.3 | 0.000001 | 0.007 |
|  | rpoC: | 0.00004 | 0.000001 | 0.001 |
|  | rpoD: | 0.04 | 0.000001 | 0.22 |
| rsd | rpoA: | 0.56 | 0.65 | 0.15 |
|  | rpoB: | 0.91 | 0.16 | 0.88 |
|  | rpoC: | 0.88 | 0.77 | 0.74 |
|  | rpoD: | 0.001 | 0.000001 | 0.00003 |
| rutR | rpoA: | 0.23 | 0.14 | 0.001 |
|  | rpoB: | 0.998 | 0.9 | 0.98 |
|  | rpoC: | 0.56 | 0.76 | 0.84 |
|  | rpoD: | 0.13 | 0.006 | 0.27 |
| sdhA | rpoA: | 0.1 | 0.58 | 0.08 |
|  | rpoB: | 0.35 | 0.1 | 0.69 |
|  | rpoC: | 0.17 | 0.77 | 0.12 |
|  | rpoD: | 0.09 | 0.72 | 0.004 |
| selD | rpoA: | 0.17 | 0.18 | 0.002 |
|  | rpoB: | 0.998 | 0.58 | 0.06 |
|  | rpoC: | 0.84 | 0.4 | 0.49 |
|  | rpoD: | 0.74 | 0.005 | 0.01 |
| soxR | rpoA: | 0.78 | 0.9997 | 0.32 |
|  | rpoB: | 0.83 | 0.998 | 0.98 |
|  | rpoC: | 0.51 | 0.98 | 0.47 |
|  | rpoD: | 0.79 | 0.91 | 0.1 |
| soxS | rpoA: | 0.29 | 0.18 | 0.00015 |
|  | rpoB: | 0.97 | 0.33 | 0.88 |
|  | rpoC: | 0.62 | 0.85 | 0.93 |
|  | rpoD: | 0.15 | 0.002 | 0.01 |
| speA | rpoA: | 0.17 | 0.12 | 0.15 |
|  | rpoB: | 0.16 | 0.57 | 0.62 |
|  | rpoC: | 0.004 | 0.001 | 0.67 |
|  | rpoD: | 0.12 | 0.26 | 0.51 |
| torR | rpoA: | 0.85 | 0.93 | 0.008 |
|  | rpoB: | 0.998 | 0.97 | 0.7 |
|  | rpoC: | 0.98 | 0.76 | 0.999 |
|  | rpoD: | 0.5 | 0.4 | 0.87 |
| usg | rpoA: | 0.05 | 0.11 | 0.00004 |
|  | rpoB: | 0.62 | 0.69 | 0.001 |
|  | rpoC: | 0.72 | 0.67 | 0.007 |
|  | rpoD: | 0.43 | 0.01 | 0.01 |
| uspG | rpoA: | 0.87 | 0.991 | 0.06 |
|  | rpoB: | 0.999 | 0.95 | 0.9998 |
|  | rpoC: | 0.49 | 0.76 | 1 |
|  | rpoD: | 0.07 | 0.84 | 0.57 |
| ydeO | rpoA: | 0.12 | 0.22 | 0.28 |
|  | rpoB: | 0.92 | 0.23 | 0.75 |
|  | rpoC: | 0.09 | 0.01 | 0.47 |
|  | rpoD: | 0.54 | 0.01 | 0.79 |
| yiaJ | rpoA: | 0.999 | N/A | 0.00025 |
|  | rpoB: | 0.58 | N/A | 0.003 |
|  | rpoC: | 0.49 | N/A | 0.0004 |
|  | rpoD: | 0.04 | N/A | 0.11 |
| zraR | rpoA: | 0.24 | 0.09 | 0.007 |
|  | rpoB: | 0.99 | 0.14 | 0.46 |
|  | rpoC: | 0.99 | 0.03 | 0.95 |
|  | rpoD: | 0.46 | 0.000001 | 0.11 |
